# Transcriptional profiling reveals that a MYB transcription factor *MsMYB4* contributes to the salinity stress response of alfalfa

**DOI:** 10.1371/journal.pone.0204033

**Published:** 2018-09-25

**Authors:** Wei Dong, Xijiang Liu, Donglei Li, Tianxue Gao, Yuguang Song

**Affiliations:** School of Life Science, Qufu Normal University, Qufu, Shandong, P.R.China; Institute of Genetics and Developmental Biology Chinese Academy of Sciences, CHINA

## Abstract

MYB transcription factors are important regulators of the plant response to abiotic stress. Their participation in the salinity stress of the key forage legume species alfalfa (*Medicago sativa*) was investigated here by comparing the transcriptomes of the two cultivars Dryland (DL) and Sundory (SD), which differed with respect to their ability to tolerate salinity stress. When challenged by the stress, DL plants were better able than SD ones to scavenge reactive oxygen species. A large number of genes encoding transcription regulators, signal transducers and proteins involved in both primary and secondary metabolism were differentially transcribed in the two cultivars, especially when plants were subjected to salinity stress. The set of induced genes included 17 *MYB* family of transcription factors, all of which were subsequently isolated. The effect of constitutively expressing these genes on the salinity tolerance expressed by *Arabidopsis thaliana* was investigated. The introduction of *MsMYB4* significantly increased the plants’ salinity tolerance in an abscisic acid-dependent manner. A sub-cellular localization experiment and a transactivation assay indicated that MsMYB4 was deposited in the nucleus and was able to activate transcription in yeast. Based on this information, we propose that the *MsMYB4* products is likely directly involved in alfalfa’s response to salinity stress.

## Introduction

Soil salinity imposes a major constraint over crop productivity; it has been estimated that almost half of the world’s irrigated land area and about 20% of arable land is affected to a varying degree by salinity[1]. Faced with an excess of salt in the soil water, most plant species suffer physiological disruption, due to one or all of ion toxicity, osmotic stress and a reduced ability to take up nutrients; the result is to inhibit plant growth and to interfere with normal development, and in extremis can lead to the plants’ inability to complete their life cycle[2]. Plants have evolved a range of morphological, physiological and biochemical strategies to cope with the stress, among which are a capacity to accumulate osmotically active compounds[3], to promote ion-selective absorption and compartmentalization[4], to neutralize reactive oxygen species (ROS)[5], to activate specific signal transduction pathways[6], and to re-program gene expression either genetically and/or epigenetically[7,8].

Alfalfa (*Medicago sativa*.L) is a prominent and widely cultivated forage legume species[9]. Salinity stress has a marked negative effect on its germination, biomass production, forage quality and nitrogen fixation[10,11]. The obligate outcrossing and allotetraploid character (2n = 4x = 32) makes it carry a large genetic diversity ranging from susceptible to tolerant to moderate salinity among different cultivars[12–14]. The adaptive mechanism of alfalfa under abiotic stress is a complex multigenic process that is not well understood. Genome scale analysis of gene expression profiles is a powerful method to make a comprehensive understanding of the changes in gene expression within plants such as cell differentiation, development and stress responses. High throughput transcriptome sequencing and digital gene expression (DEG) tag profiling are efficient and cost effective methods for characterizing non-model organisms without the need for a reference genome [15,16]. Based on this technique, some studies have been reported in monitoring gene expression under abiotic stress in alfalfa[17–19]. These data indicated that the transcriptional changes of genes mainly involved in antioxidant, energy metabolism, photosynthesis, ionic equilibrium, signal transduction and phytohormone biosynthesis in responding to salinity, drought, freezing/heat and heavy metal stresses[20–22].

Transcription factors have emerged as key regulators of the general plant response to abiotic stress. The large class of MYB (v-myb avian myeloblastosis viral oncogene homolog) transcription factors are characterized by the presence of certain repeated ~52 residue motifs in their DNA binding region. The mature protein forms three α helices, two of which participate in the formation of a helix–turn–helix (HTH) fold[23]. Based on the number and placement of the MYB domain(s), four major MYB groups (1R through 4R) have been recognized. The 1R proteins are thought to be largely involved in phosphate-sensing, reproductive growth and circadian clock control[24]; the 2R proteins in primary and secondary metabolism, signal transduction, the determination of cell fate identity, development and the stress response[25–28]; the 3R proteins are largely associated with the control of cell cycling[29]; but little is known regarding the function of the 4R proteins in plants[23]. The R2R3-MYB proteins are a family of plant-specific, highly abundant plant transcription factors. The extensive characterization of R2R3 MYBs has frequently implicated them as forming a part of the regulatory system underlying salinity tolerance, not just in the model plant species *Arabidopsis thaliana*, but also in a number of crop species, including rice, maize, soybean and wheat[30–34]. However, there is still little information about the role of MYB transcription factors in the salinity response of alfalfa. The present study focuses on the identification of MYB TFs that involved in the short-term and more sustained responses of salinity stress by contrasting the salt tolerant alfalfa cultivar Dryland (DL) transcriptome with that of salt sensitive Sundory (SD) using DGE profiling method. A R2R3-MYB TF gene *MsMYB4* has been characterized. The gene was shown to be inducible by salinity and ABA treatment, and its constitutive expression in *A*. *thaliana* demonstrated to enhance the plant’s tolerance to salinity stress.

## Materials and methods

### Plant materials and growing conditions

The germination rate of the seed of the two cultivars (the salinity tolerant DL and the salinity sensitive SD) was determined following standard practices. In brief, 100 seeds of each cultivar was laid on filter paper moistened with either distilled water or 150 mM NaCl, and held at 25 °C. Germination rates were assessed after three days, when the length of the radicle emerging from the majority of the seeds had reached at least 1 cM. To assess biomass accumulation, 25 uniform seedlings were grown in a 10 cM diameter pot filled with a vermiculite:peat (2:1) mixture for two weeks under 25 °C day/20 °C night, 14 h photoperiod, and 50% relative humidity condition, then they were exposed to 200 mM NaCl for another two weeks. At the end of this period, both the length of the shoot and its fresh weight were measured from a 100 seedlings per cultivar. For the physiological assays, and the preparation of RNA used for both the RNA-seq libraries and for quantitative real time PCR (qRT-PCR) assays, seedlings were soil grown as above, but after the two weeks period (prior to the salinity treatment), they were exposed to either 200 mM NaCl, 20% w/v PEG6000 or 100 μM abscisic acid (ABA). Each treatment was replicated three times. Plant tissue required for RNA extraction was snap-frozen in liquid nitrogen and stored at −80°C until required.

### Physiological assays

The proline content of root tissue was determined following Qiu et al. (2017)[35], while its malondialdehyde (MDA), soluble sugar and H_2_O_2_ content, along with the activity of the enzymes peroxidase (POD), superoxide dismutase (SOD), glutathione peroxidase (GPX), catalase (CAT) and ascorbate peroxidase (APX), were assessed using commercially available kits (Jiancheng, Nanjing, China) with triple replicates.

### RNA-seq procedure

RNA sequencing tagged libraries were constructed from root mRNA extracted from either salinity-stressed (both 1 h and 24 h exposure time) DL and SD seedling roots, as well as from non-stressed ones. A 1.5 μg aliquot of mRNA per sample was used as input material. Sequencing libraries were generated using an NEBNext^®^ Ultra^™^ RNA Library Prep Kit for Illumina^®^ (New England Biolabs) following the supplier’s protocol, and index codes were added to attribute sequences to their source sample. Clustering of the index-coded samples was performed with a cBot Cluster Generation System using TruSeq PE Cluster Kit v3-cBot-HS (Illumina, San Diego, USA) according to the manufacturer’s instructions. The libraries were sequenced on an Illumina Hiseq platform by Novogene Co. (Beijing, China). Raw reads were filtered for adaptor sequences, those containing poly-N sequences and low quality reads. The subsequent transcriptome assembly was accomplished using the min_kmercov parameter set to 2 and all other parameters set to their default value, following Grabherr et al.(2011)[36]. Gene function was annotated based on the following databases: Nr (NCBI non-redundant protein sequences); Nt (NCBI non-redundant nucleotide sequences); Pfam (Protein family); KOG/COG (Clusters of Orthologous Groups of proteins); Swiss-Prot (A manually annotated and reviewed protein sequence database); KO (KEGG Ortholog database); GO (Gene Ontology).

### Differential transcription and Gene Ontology (GO) enrichment analysis

Prior to differential gene expression analysis, for each sequenced library, the read counts were adjusted by edgeR program package through one scaling normalized factor. Differential expression analysis of two samples was performed using the DEGseq (2010) R package. P-value was adjusted using q value. qvalue<0.005 & |log2^(foldchange)^|>1 was set as the threshold for significantly differential expression. Gene Ontology (GO) enrichment analysis of the differentially expressed genes (DEGs) was implemented based wallenius non-central hyper-geometric distribution[37], which can adjust for gene length bias in DEGs.

### Identification of MYB sequences and their phylogenetic analysis

The assembled transcriptome sequences were submitted for the identification of transcription factors to the iTAK program (itak.feilab.net). The alafalfa MYB sequences were aligned with the set of *A*. *thaliana* MYB protein sequences downloaded from phytozome (phytozome.jgi.doe.gov) using ClustalX software (www.clustal.org). The subsequent phylogenetic analysis relied on the neighbor-joining method, as implemented in MEGA v6.0 software (www.megasoftware.net) and a bootstrap analysis was applied, based on 1,000 replicates.

### Transcription profiling using qRT-PCR

RNA was extracted from the roots of control and treated seedlings using the TRIzol reagent (Transgene, Beijing, China). A 1 μg aliquot of DNase-digested total RNA was used to synthesize the cDNA first strand using a First-Strand cDNA Synthesis Kit (Transgene, Beijing, China) following the manufacturer’s protocol. The subsequent PCRs were performed using a SYBR Premix Kit (Vazyme, Nanjing, China). Each 10 μL reaction contained 5 μL SYBR Green mix, 3 μM of each primer and 1 μL cDNA. The reactions were initially denatured (94°C/4 min), then subjected to 40 cycles of 95°C/10 s, 60°C/30 s. The alfalfa *Actin2* gene (GenBank accession number JQ028730) was used as the reference. Relative transcription abundances were calculated using the 2^-ΔΔCt^ formula, following Livak and Schmittgen (2001) [38]. Each of the three biological replicates was supported by three technical replicates. Relevant primer sequences are listed in S7 Table.

### Sub-cellular localization of MsMYB4 and transactivation assay of MsMYB4 in yeast

The coding sequence of *MsMYB4* (lacking its stop codon) was fused to the N terminus of *GFP* represented in the construct p*CaMV35S*::*MsMYB4*. The fused vector was transformed into *A*. *thaliana*, and transgene homozygous progenies were selected. The sub-cellular localization of GFP activity was monitored using a confocal laser scanning microscope (Leica) equipped with a 488 nm filter. To characterize the transactivation of *MsMYB4* in yeast, the full length coding sequence of *MsMYB4* was amplified and cloned into *pGBKT7* vector, and then transformed into the yeast strain AH109 (*MATa*, *trp1–901*, *leu2–3*, *112*, *ura3–52*, *his3–200*, *gal4*Δ, *gal80*Δ, *LYS2*::*GAL1*_*UAS*_ *-GAL1*_*TATA*_ *-HIS3*, *GAL2*_*UAS*_ *-GAL2*_*TATA*_ *-ADE2*, *URA3*::*MEL1*_*UAS*_ *-MEL1*_*TATA*_ *-lacZ*, *MEL1*). The empty *pGBKT7* (BD) and fusing the GAL4 vectors were used as negative and positive controls, respectively. The transactivation activity was evaluated according to the growth on SD/−Trp and SD/−Trp–His–Ade media at 30 °C for 3 days.

### Stress treatment and phenotypic analysis of transgenic plants

Seeds harvested from transgene homozygous plants (OE lines) and from lines carrying a empty vector (VC line) were surface-sterilized and plated on solidified medium containing half strength Murashige and Skoog (MS) salts. The plates were held at 4°C in the dark for two days, then removed to a chamber delivering a 16 h photoperiod and a constant temperature of 22°C. After three days, the seedlings were transferred to a fresh plate containing the same medium supplemented with various concentrations of either NaCl or of ABA, where they were allowed grow for a further ten days. Each experiment was represented by three replicates. To determine the ability of *A*. *thaliana* seeds to germinate in the presence of NaCl or ABA, 80~100 seeds of each of wild type, the empty vector line and the two *MsMYB4* constitutive expressor lines were surface-sterilized and plated on solidified medium containing half strength MS salts supplemented by 1% (w/v) sucrose plus a variable concentration of NaCl or ABA. The plates were held at 4°C in the dark for two days, then removed to the light at 21°C. Germination was deemed as successful when the radical became visible, and was scored at various time points.

## Results

### The phenotypic and physiological response of alfalfa to salinity stress

**Fig 1:**
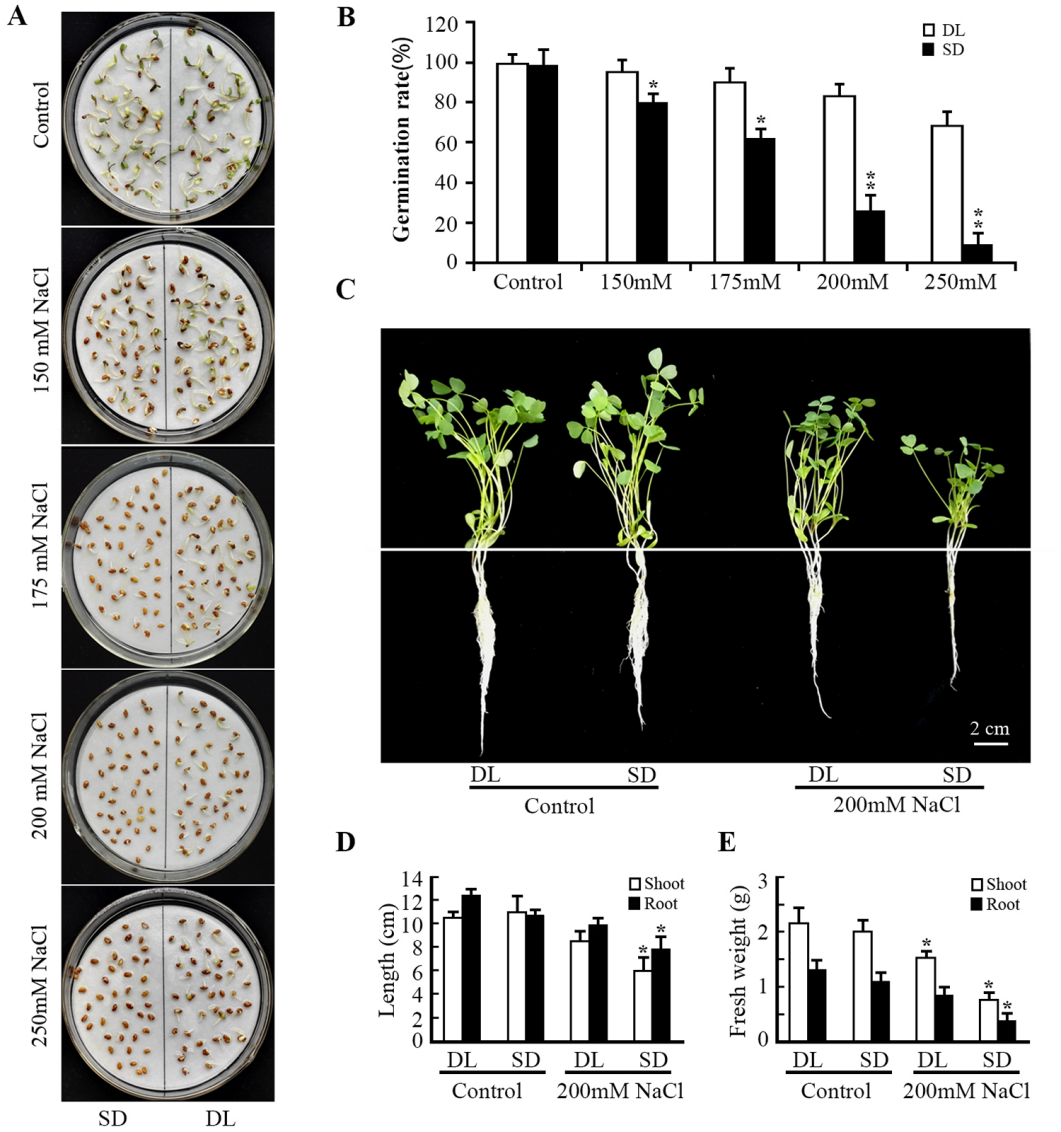
The effect of salinity on the germination rate and seedling growth of the two contrasting alfalfa cultivars DL and SD. (A) and (B) Seed germination rate measured after four days in the absence and presence of salinity stress. (C) the appearance of seedlings grown hydroponically in the presence of either 0 mM or 200 mM NaCl. (D) and (E) the effect of salinity stress (200 mM NaCl for 14 days) on (D) root elongation, (E) shoot fresh weight (the average weight of ten seedlings). Data given in the form mean±SE. **: means differed significantly at P <0.01.

**Fig 2:**
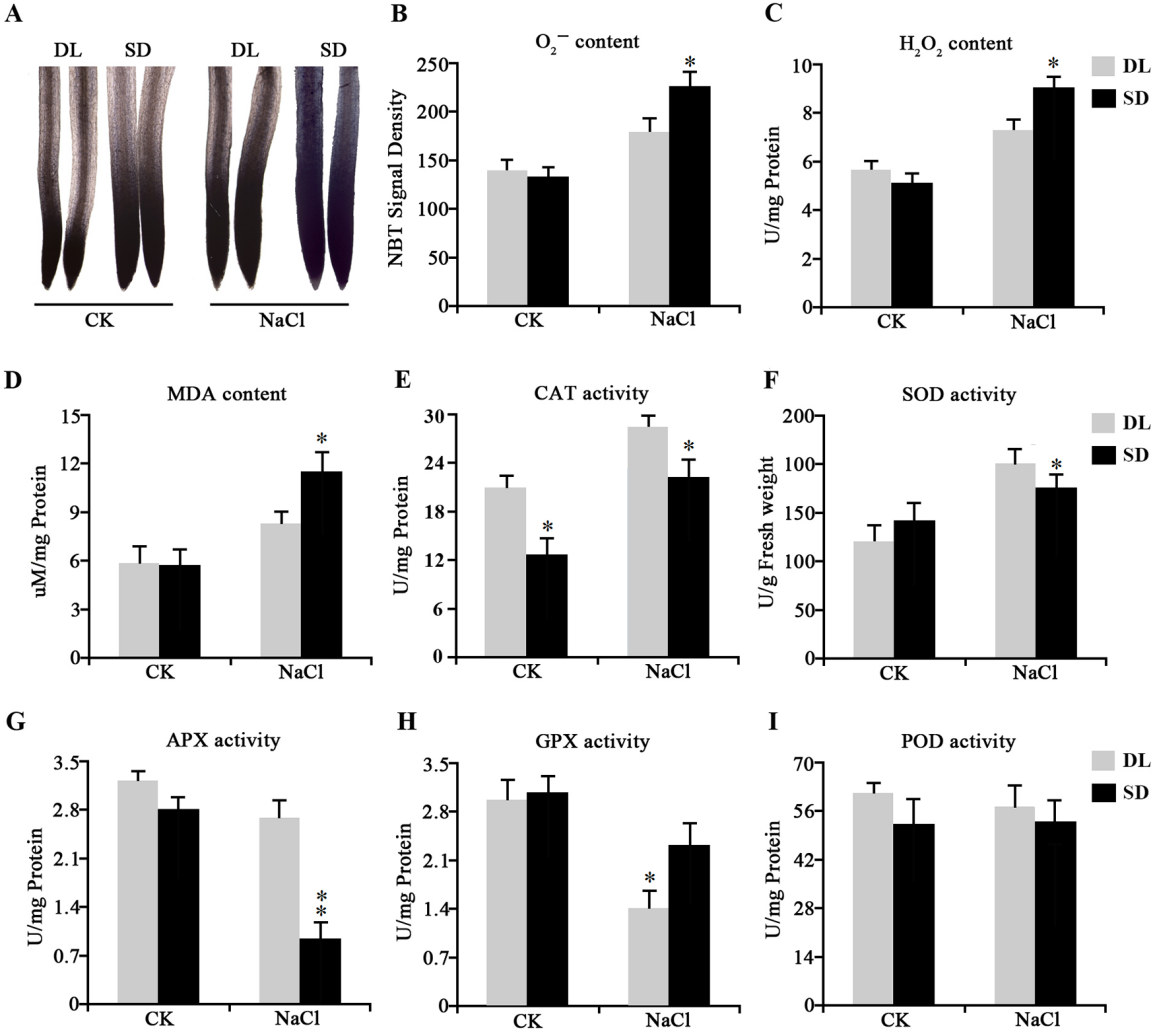
The ROS content and activity of key ROS scavenging enzymes in the roots of DL and SD seedlings either in the absence or presence of NaCl. (A) Root superoxide content. (B) relative superoxide contents calculated from mean pixel densities of the images shown in (A). (C)-(I) the activity of (C) H_2_O_2_, (D) MDA, (E) catalase (CAT), (F) superoxide dismutase (SOD), (G) ascorbate peroxidase (APX), (H) glutathione peroxidase (GPX), (I) peroxidase (POD). test: Data given in the form mean±SE. * and **: means differed significantly at, respectively, P <0.05 and <0.01.

A comparison of the germination rate and the seedling growth made between DL and SD confirmed that they differed markedly with respect to salinity tolerance. The germination, growth of the seedling and the roots were similar between DL and SD when they were grown in the absence of salinity stress (Fig 1A and 1C). However, DL’s germination rate was reduced from ~100% to ~72%, while that of SD fell from ~100% to ~12% under condensation of NaCl from 0 mM to 250 mM (Fig 1B). The stress imposed by salinity also had a differential effect on the cultivars’ shoot and root length and fresh weight. After an exposure to 200 mM NaCl for two weeks, shoot and root lengths were reduced by, respectively ~16% and ~11% for DL, but by ~45% and ~19% for SD (Fig 1D); meanwhile both shoot and root fresh weight suffered a reduction of, respectively ~37% and ~40% in DL, but of ~70% and ~63% in SD (Fig 1E). A comparison of the two cultivars’ accumulation of MDA and the level of activity of the major ROS scavenging enzymes showed that in the absence of salinity stress there was little difference between DL and SD, while in the presence of stress, DL accumulated less ROS (superoxide and H_2_O_2_) than did SD (Fig 2A and 2B); although the H_2_O_2_ and MDA content of both cultivars was raised by the imposition of the stress, the increase was less marked in DL (Fig 2C and 2D). The activity of both CAT and SOD was consistently higher in DL than in SD (Fig 2E and 2F). Both APX and GPX activity were inhibited in DL and SD by the salinity stress, but that of APX was lower in SD than in DL, and vice versa for GPX (Fig 2G and 2H). The POD content in the two varieties were not significant changed under control and salt stress (Fig 2I).

### The root transcriptome of DL and SD seedlings exposed to salinity stress

**Fig 3:**
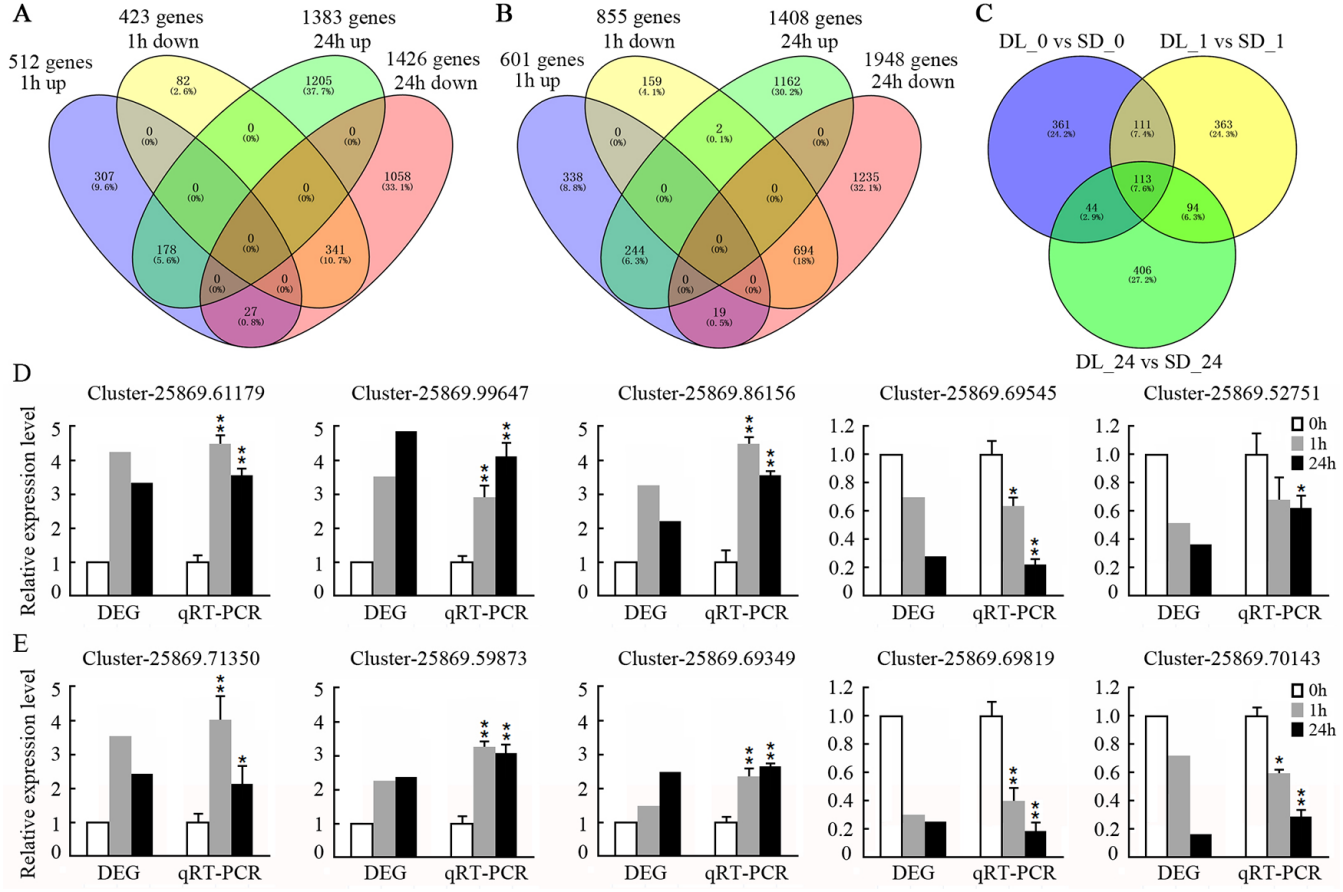
The root transcriptome of seedlings exposed to salinity for either 1 h or 24 h. (A) Genes up- or down-regulated by salinity stress in DL. (B) Genes up- or down-regulated by salinity stress in SD. (C) DEGs identified by comparing the transcriptomes of DL and SD seedlings exposed to either no salinity (DL_0 *vs* SD_0), or salinity stress for either 1 h (DL_1 *vs* SD_1) or 24 h (DL_24 *vs* SD_24); (D)-(E) The expression level analyzed by RNA-seq and qRT-PCR of five genes in (D) DL roots exposed to salinity stress for 0 h, 1 h, 24 h and five in (E) SD roots exposed to salinity stress for 0 h, 1 h and 24 h.

A total of 280,546,400 clean reads were recovered from the set of control (non-stressed) and salinity-stressed RNA-seq libraries made from DL and SD seedling roots (https://www.ncbi.nlm.nih.gov/Traces/study/?acc=SRP158757&go=go, accession numbers: SRR7751381 to SRR7751386); the number of sequences per sample ranged from 40,872,344 to 58,609,528. About 70% of these sequences were mappable onto reference sequences (S1 Table). The abundance of 3,198 transcripts was altered by the stress treatment in DL and that of 3,853 in SD (Fig 3A and 3B). To verify whether the DGE output represented the true variation of the transcripts, ten genes (five from the RNA-seq libraries made from DL and another five from SD) were randomly chosed for the qRT-PCR amplification. The results showed that their expression profiles were highly consistent between the RNA-seq platform and the qRT-PCR method (Fig 3D and 3E). The results were clearly showed that the qRT-PCR data were consistent with the DEG output, suggesting that RNA-seq results were highly reliable.

In plants grown under non-stressed conditions, the transcript abundance of 629 genes differed(P< 0.005, |fold change| >2) between DL and SD (Fig 3C). The effect of a 1 h exposure to salinity stress was to alter the abundance of 935 transcripts in DL (512 increased, 423 decreased) and that of 1,456 (601 increased, 855 decreased) in SD (Fig 3A and 3B; S2 and S3 Tables). A GO analysis of these genes revealed that 24 GO categories were over- represented in DL and 16 in SD (P <0.01, FDR <0.05) (S4 Table). In both DL and SD, the most frequently represented categories were “oxidation/reduction”, “metabolic processes” and “stress response”. Unique to DL were genes assigned to the categories “nucleic acid synthesis” (e.g., GO:0019219 and GO:2001141) and “transcriptional processes” (GO:0006355). With respect to the category “oxidation/reduction” (GO:0055114), there were 169 re-programmed genes in DL but only 81 in SD (S4 Table). Following the 24 h exposure to salinity, 2,809 (1,383 increased, 1,426 decreased) and 3,356 (1,408 increased, 1,948 decreased) DEGs were identified in, respectively, DL and SD (Fig 3A and 3B; S2 and S3 Tables). These genes were classified into 96 (DL) and 86 (SD) GO categories. The over-represented categories were similar for the two cultivars; the majority involved metabolism, the stress response and ion transport (S4 Table). The GO categories “cell wall organization” (GO:0071555), “cell wall biogenesis” (GO:0042546), “photosynthesis” (GO:0009765) and “hormone metabolic processes” (GO:0042445) were over-represented in DL but not in SD in the 24 h salinity treatments (S4 Table). Of the set of DEGs, 178 were up- and 341 down-regulated in DL in both salinity treatments, and the equivalent numbers for SD were, respectively, 244 and 694 (Fig 3A and 3B). The transcript abundance of 681 genes differed between DL plants exposed to either 1 h or 24 h of stress, while the equivalent number for SD was 657 (Fig 3C). In addition, the expression of 113 genes were altered between DL and SD both under the control (0 h) and salinity stress (1 h, 24 h) condition (Fig 3C).

### The identification of differentially transcribed genes encoding MYB transcription factors

**Fig 4:**
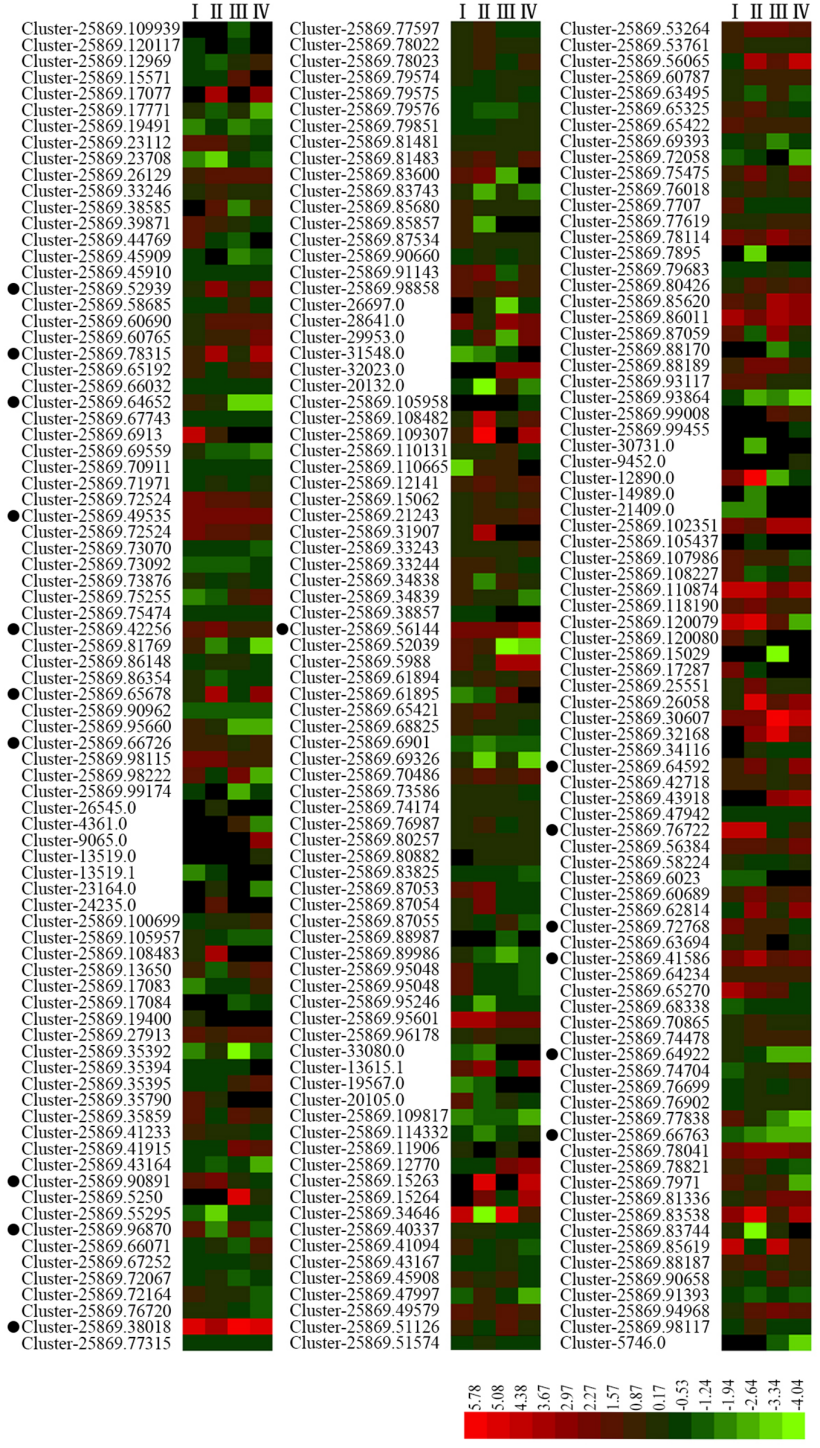
The expression patterns of MYBs under control and salt stress. Genes were identified from the (non-stressed) and salinity-stressed RNA-seq libraries made from DL and SD seedling roots which contain a core sequence of the MYB family genes. I-IV: The expression pattern of the genes under the comparison of I: DL_1 *vs* DL_0, II: DL_24 *vs* DL_0, III: SD_1 *vs* SD_0, IV: SD_24 *vs* SD_0. Genes marked with the black dots were the 17 differentially expressed (|fold change| >2, q-value<0.1) MYBs identified from the above comparison.

In order to identify the MYB transcription factors that involved in salinity stress, the DEG were mapped into plant transcription factor database, then selected by hmmscan based on the iTAK pipeline. As a result, 247 DEGs annotated in the database contain a core sequence of the MYB family genes (Fig 4; S5 Table). Among them, a total of 17 of the genes which were differentially transcribed (|fold change| >2, q-value<0.1) as a result of exposure to salinity stress in either DL and/or SD contained a core sequence characteristic of the *MYB* gene family: these were allocated the gene symbols *MsMYB1* through *MsMYB17* (Fig 4; S5 Table). In DL, 12 of these genes were up-regulated (two in the 1 h exposure treatment, seven in the 24 h treatment and three in both treatments) and 1 was down-regulated in both treatments; In SD, 8 of the genes were up-regulated (two in the 1 h treatment, four in the 24 h treatment and two in both treatments) while 2 were down-regulated (one in the 1 h treatment and one in both treatments). 2 of the genes were up-regulated in both cultivars and 1 was down-regulated in both treatment of DL and SD (Fig 4; S5 Table). The diversity expression pattern of these genes suggesting that they may be involved in salt stress responses at different stages in DL and SD.

### The isolation and sequence analysis of the *MsMYBs*

**Fig 5:**
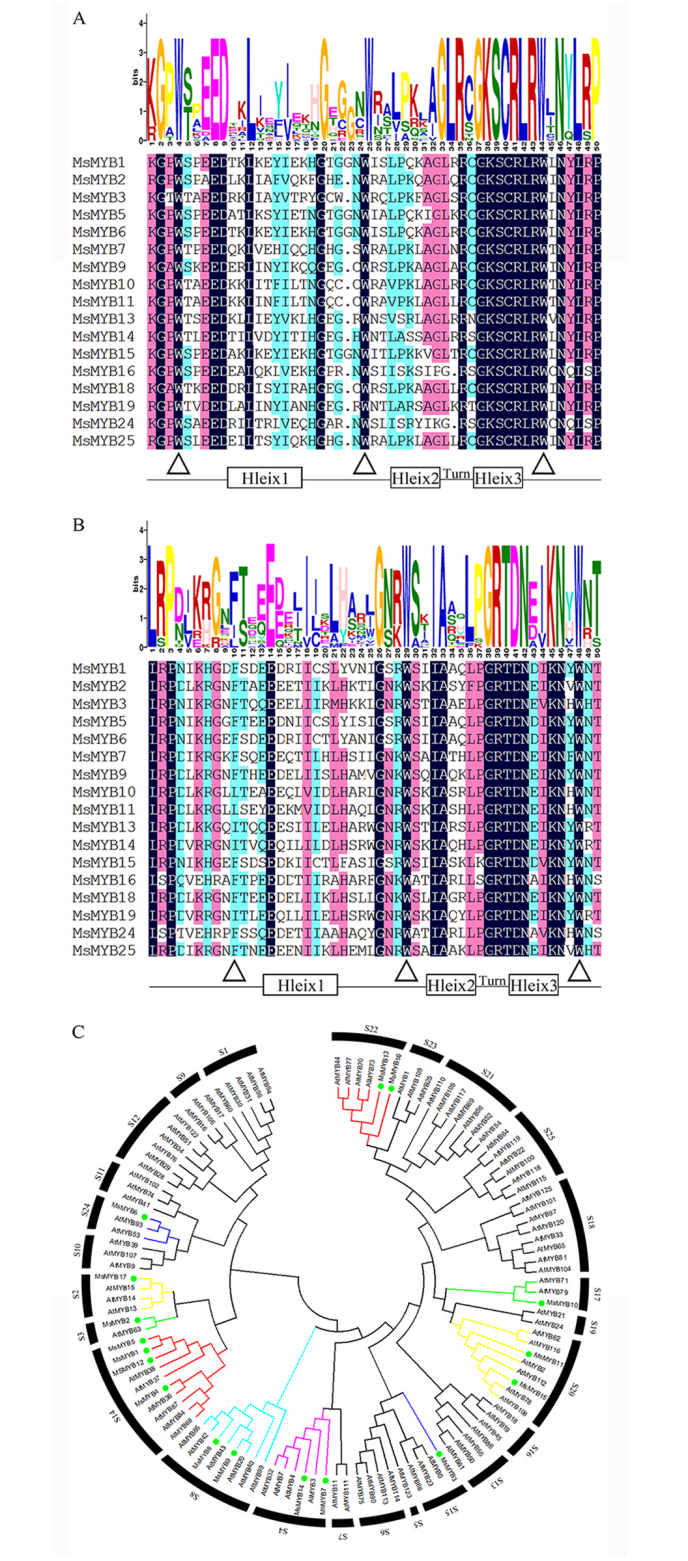
Predicted domains phylogenetic analysis of MsMYBs. (A) The R2 and (B) the R3 domains. The *y* axis (measured in bits) depicts the overall height of the stack and indicates the level of sequence conservation at that position. The symbol height within the stack indicates the relative frequency of each residue at that position. (C) Phylogenetic analysis of MsMYBs with *A*. *thaliana* MYB transcription factors. The *A*. *thaliana* sequences were retrieved from the Phytozome database. The level of support for the various nodes was estimated using 1,000 bootstrap replicates.

As only partial sequences were recovered from the transcriptome database and there was no reference genome sequence for alfalfa, primers were designed to amplify the full coding sequences (CDS) of each of the 17 genes, based on the sequence of their probable orthologs in *M*. *truncatula* and finally their CDS were all isolated (All the CDS sequence of the 17 *TFs* were listed in S6 Table). The length of the predicted MsMYB polypeptides ranged from 242 (MsMYB16) to 349 (MsMYB4) residues, their predicted molecular weights from 26.5 kDa (MsMYB16) to 39.3 kDa (MsMYB4), and their predicted pIs from 4.66 (MsMYB2) to 9.41 (MsMYB7). Their MYB domain (R unit) sequences were highly conserved: all of the predicted gene products included two R units (S1 Fig). The R2 repeats harbored three well conserved tryptophan residues at positions 4, 25, and 44 (Fig 5A); tryptophan residues were well conserved at positions 29 and 48 of the R3 repeats (Fig 5B). Other conserved residues present were G2, E8, D9, L12, G20, R36, G38, K39, S40, C41, R42, L43 and R44 in the R2 repeat (Fig 5A), and L1, P3, E14, G26, I32, A33, G38, R39, T40, D41, N42, K45 and N46 in the R3 repeat (Fig 5B). To infer the evolutionary relationships of these *MsMYBs*. A phylogenetic tree was constructed between the obtained 17 *MsMYBs* and 97 known R2-R3MYBs from *Arabidopsis* (Fig 5C). All the 114 MYB were classified into 25 subgroups. Meanwhile, 17 MsMYBs proteins were divided into 10 subgroups (S2, 3, 4, 8, 14, 15, 17, 20, 22 and 24) according to their orthologous MYBs from *Arabidopsis*. This result suggests the existence some closely related orthologous MYBs between alfalfa and *Arabidopsis*. This provides an important reference for the analysis and prediction of the functions of these TFs in alfalfa.

### The transcriptional response of *MsMYBs* to salinity stress

**Fig 6:**
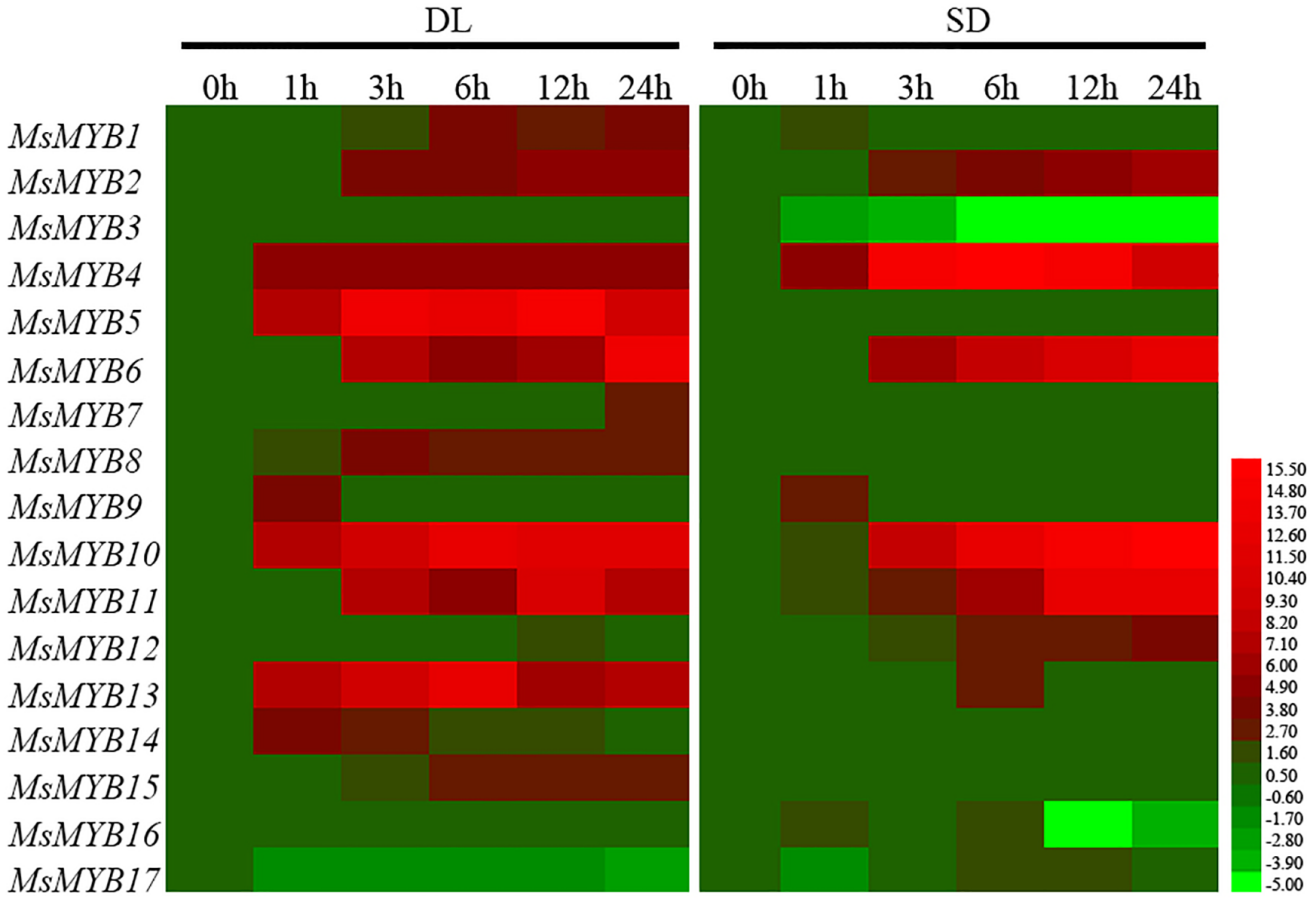
Transcription profiling of the set of *MsMYB*s in plants grown either in the absence or presence of salinity stress.

A qRT-PCR assay was used to characterize the temporal response of each of the *MsMYB*s to salinity stress in both DL and SD seedlings (Fig 6). *MsMYB9* was shown to be induced after a 1 h exposure to the stress in both DL and SD, while *MsMYB7* was induced much later (24 h). The abundance of *MsMYB2/6/10/11* transcripts increased gradually over the period 1–24 h, while *MsMYB3* was down-regulated. *MsMYB4/13* transcription peaked at 6 h then dropped away. *MsMYB1/2/4/6/10/11* were up-regulated by the stress in both cultivars, while *MsMYB5/7/8/13/14/15* were up-regulated only in DL and *MsMYB12/17* only in SD (Fig 6).

#### 3.6 The constitutive expression of *MsMYB4* in *A*. *thaliana* enhances salinity tolerance

**Fig 7:**
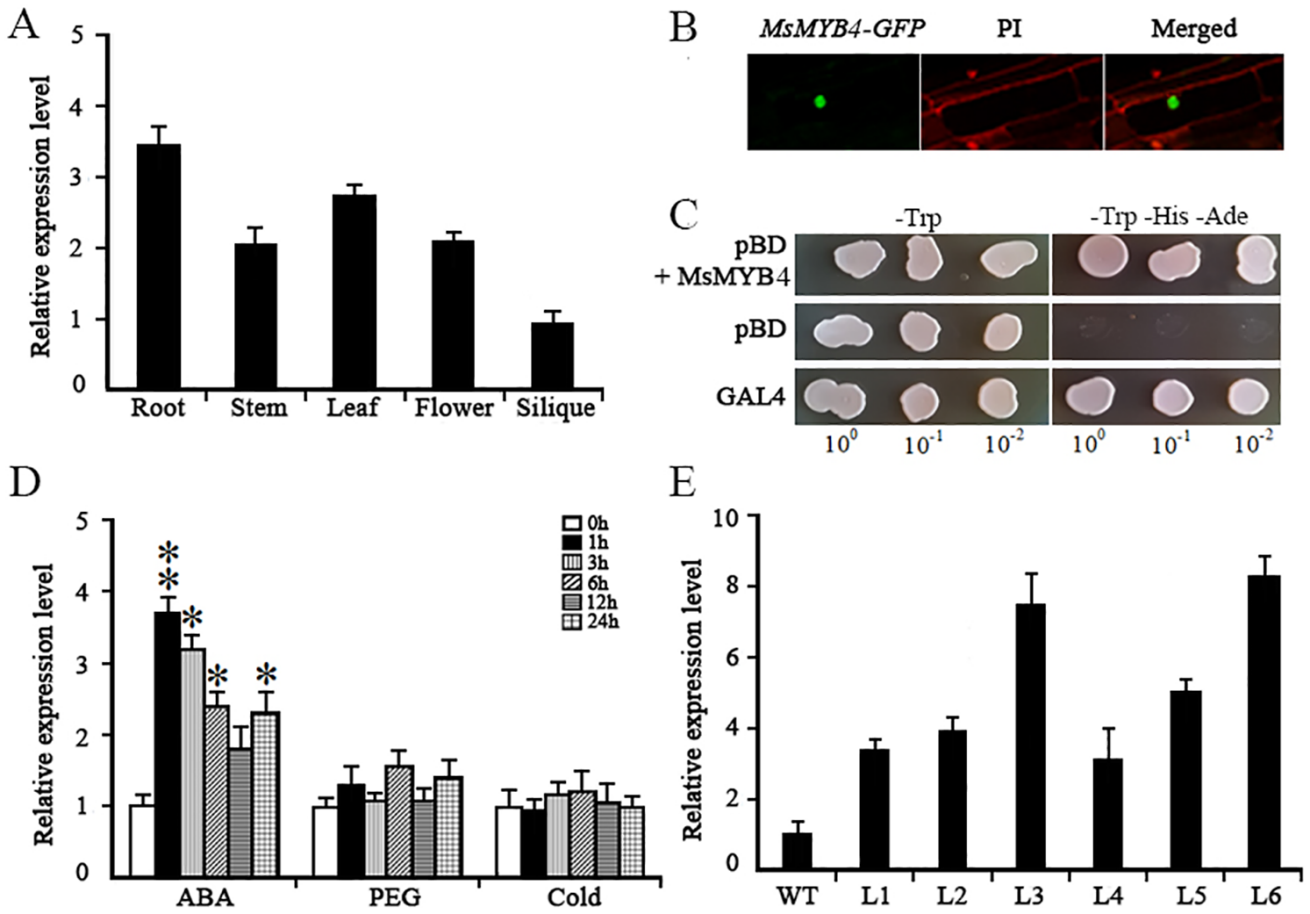
The sub-cellular localization and transcriptional activation capacity of *MsMYB4*, along with its transcriptional response to diverse abiotic stress factors. (A) Variation in the transcription of *MsMYB4* in different tissue of the alfalfa plant. (B) the sub-cellular localization of *MsMYB4* expression. (C) the transactivation activity of *MsMYB4* in yeast. Transformed yeast cells were selected on both SD-Trp and SD-Trp-His-Ade media. (D) the transcriptional response of *MsMYB4* to exposure to ABA, PEG (moisture deficiency) and low temperature. (E) Variation in the abundance of *MsMYB4* transcript among six independent transgene homozygous *A*. *thaliana* lines.

To determine whether these *MsMYBs* involved in salt tolerance, the full CDS of each of the 17 *TFs* were fused into the *35S*:*GFP*:*NOS*:*1300* vector (The fragment was inserted between p*CaMV35S* and *GFP*) by using the ClonExpress ^®^ II One Step Cloning Kit (Vazyme, Nanjing, China) then transferred into *Arabidopsis*. A line harboring an empty vector (VC line) was also constructed served as the negative control. The homozyous lines were selected then salt tolerance ability of these transgenic *Arabidopsis* with a ectopic expression of above genes were analyzed. As a result, among the *A*. *thaliana* lines over-expressing the various *MsMYB*s, the line harboring *MsMYB4* exhibited a performance superior to that of wild type with respect to root growth under salinity stress. Within alfalfa itself, *MsMYB4* was transcribed most strongly in the root and leaf (Fig 7A), and the site of GFP activity arising from the constitutive expression of a *MsMYB4-GFP* fusion transgene (Fig 7B) suggested that MsMYB4 was a nuclear-localizing protein, consistent with its predicted function as a transcription factor. As expected therefore, the growth of transformants carrying *pGBKT7-MsMYB4* on selective medium (SD/−Trp) and (SD/−Trp–His–Ade) indicated the *MsMYB4* protein has transcriptional activity, the *pGBKT7*-*GAL4* and empty *pGBKT7* were used as positive and negative control, respectively (Fig 7C). *MsMYB4* was found to be inducible by treating alfalfa seedlings with ABA (Fig 7D), implying that the activity of MsMYB4 is dependent on ABA.

**Fig 8:**
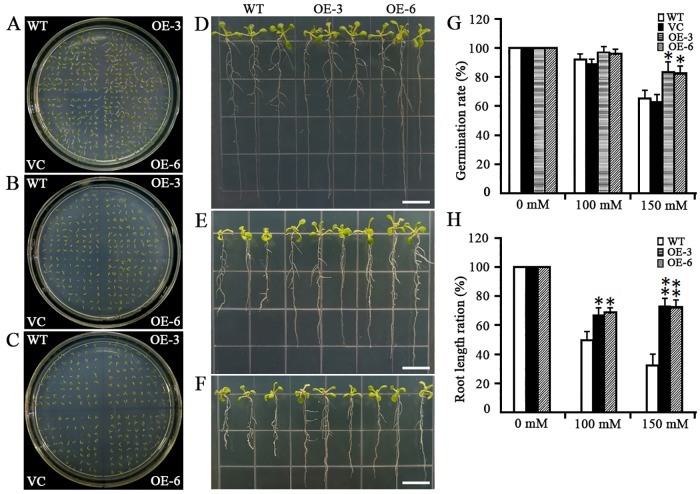
The response to salinity stress of *A*. *thaliana* plants constitutively expressing *MsMYB4*. (A)-(C) Germination rate of seeds exposed to (A) 0 mM, (B) 100 mM, (C) 150 mM NaCl. (D)-(F) the effect of salinity stress on control and transgenic lines exposed to (D) 0 mM, (E) 100 mM, (F) 150 mM NaCl for ten days, (G) quantification of the germination rate of the materials illustrated in (A-C). (H) quantification of the root length of the seedlings illustrated in (D-F). VC: transgenic *A*. *thaliana* carrying an empty vector, OE3 and OE6: transgenic *A*. *thaliana* lines constitutively expressing *MsMYB4*. Bar: 2 cm. Means derived from the measurement of ten seedlings; *and**: means differed significantly at, respectively, P <0.05 and <0.01.

**Fig 9:**
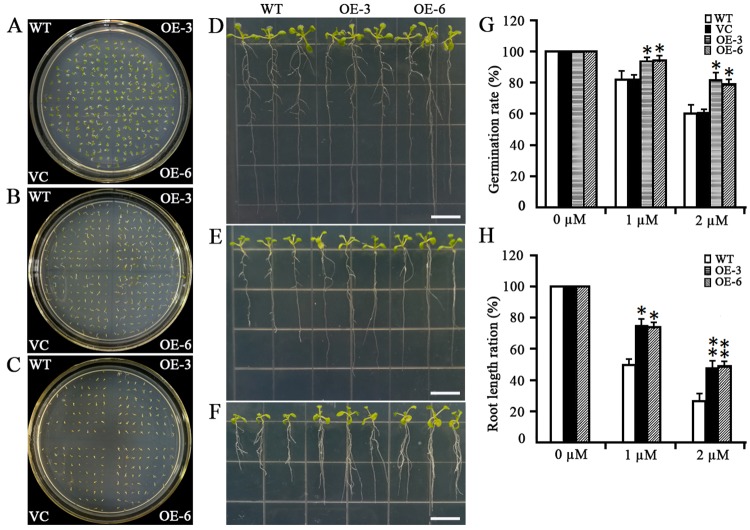
The response of *A*. *thaliana* plants constitutively expressing *MsMYB4* to the presence of exogenously supplied ABA. (A)-(C) The germination rate of seeds in the presence of (A) 0 μM, (B) 1 μM, (C) 2 μM ABA. (D)-(F) the effect of the ABA treatment on materials exposed for ten days to (D) 0 μM, (E) 1 μM, (F) 2 μM ABA. (G) quantification of the germination rate of the materials illustrated in (A-C). (H) quantification of the root length of the seedlings illustrated in (D-F). (D-F). VC: transgenic *A*. *thaliana* carrying an empty vector, OE3 and OE6: transgenic *A*. *thaliana* lines constitutively expressing *MsMYB4*. Bar: 2 cm. Means derived from the measurement of ten seedlings; *, **: means differed significantly at, respectively, P <0.05 and <0.01.

Inspection of the variation in the abundance of *MsMYB4* transcript among six independent transgene homozygous *A*. *thaliana* lines resulted in the selection of two of these (L3 and L6) for the further experiments (Fig 7E). Under non-saline conditions, the germination rate of seed produced by wild type *A*. *thaliana* plants did not differ from that of seed produced by either of the two selected transgenic plants (Fig 8A–8C). However, in the presence of 150 mM NaCl, 60% of the wild type seeds germinated, whereas 84.4% of seed of the transgenic line OE-3 and 82.3% of the OE-6 were able to germinate (Fig 8C and 8G). When seedlings were grown on a medium containing either 100 mM or 150 mM NaCl, both of the *MsMYB4* constitutive expressors displayed better root growth than was possible for wild type seedlings (Fig 8D–8F and 8H). The inclusion of ABA in the medium resulted in a smaller fall in the germination rate of the *MsMYB4* constitutive expressors than was experienced by wild type seed (Fig 9). In the presence of 1 μM ABA, the germination rate of wild type seed was ~80%, while that of the seed of both independent *MsMYB4* transgenics OE-3 and OE-6 was around 95% (Fig 9A, 9B and 9G). When the concentration of ABA was raised to 2 μM, the germination rate of wild type seed fell to around 60%, while that of the transgenic seed fell to around 80% (Fig 9C and 9G). In addition, the extent of cotyledon opening was higher in the transgenic than in the wild type seedlings (Fig 9B and 9C). The roots of the transgenic seedlings were better able to grow in the presence of ABA than those of the wild type seedlings (Fig 9D–9F): thus the length of the transgenics’ roots in the presence of 2 μM ABA was about 45% of that achieved in the absence of ABA, whereas those of wild type seedlings only grew to 23% of the length of their control seedlings (Fig 9H). In summary, these findings lead to the reasonable speculation that *MsMYB4* plays an important role in salinity stress response, may be through a ABA-dependent manner.

## Discussion

### DL has a more effective ROS scavenging ability than SD

ROS (primarily superoxide, peroxide and hydroxyl), which comprise partially reduced or activated forms of oxygen, are unavoidable byproducts of aerobic metabolism; when they are accumulated as a result of stress, they can damage membranes, proteins, RNA and DNA[39]. Plants have developed the ability to neutralize ROS in a number of ways, but the balance between SOD and APX or CAT activity is particularly important for determining the steady-state level of both superoxide and peroxide[40]. Inspection of the likely function of many of the genes which were up-regulated in salinity-challenged DL showed that they were involved in oxidation/reduction, the oxidative response and the activation of peroxidase (S4 Table). Thus, while in DL, the abundance of 169 transcripts categorized as genes involved in oxidation/reduction (GO:0055114) was increased within 1 h of the imposition of salinity and of 404 within 24 h, the equivalent numbers in SD were only 81 and 231, respectively (S4 Table). When ROS scavenging enzyme activities were measured, those of CAT, SOD and APX were higher in salinity-challenged DL than in salinity-challenged SD (Fig 2E, 2F and 2G). The implication was that these three enzymes all play a prominent role in ROS scavenging. As well as DL might have a much powerful ROS-scavenging ability than SD, which also may help to explain the better performance of DL under salinity stress.

### The contrasting primary and secondary metabolism of DL and SD

Stress affects many primary and secondary plant metabolic processes. As is the case for many plants, salinity inhibited the growth of both of the alfalfa cultivars, although its impact on SD was greater (Fig 1); meanwhile many genes involved in carbohydrate metabolism were re-programmed (S2A–S2C Fig). The capacity to construct cell walls is an important requirement for plant growth, and the analysis showed that a larger number of genes involved in this process were up-regulated in salinity-challenged DL seedlings than in salinity-challenged SD ones (S2B and S2C Fig). The synthesis of the secondary metabolites terpenoids, phenolic alkaloids and flavonoids contributes strongly to the regulation of plant development. R2R3 MYB transcription factors control the production of flavonoids, which can act as anti-oxidants and thus are a key component of the plant stress response[41]. A number of genes involved in flavonoid metabolism were induced by salinity stress in DL, but this was not the case in SD (S4 Table), consistent with the discovery that R2R3 MYB transcription factors contribute significantly to inducing isoflavonoid synthesis[42]. The suggestion is therefore that the stronger capacity of DL seedlings to accumulate flavonoids explains at least a part of their advantage over SD in the context of maintaining their growth in the face of salinity stress.

### The multiple potential functions of the MsMYBs

Numerous R2R3 MYB proteins have to date been characterized as being involved in the control of plant primary and secondary metabolism, cell fate and identity, development and the response to both biotic and abiotic stress[43]. The 17 salinity induced *MsMYBs* identified here fell into the ten (of 25) recognized MYB subgroups, namely S2-4, 8, 14, 15, 17, 20, 22 and 24; specifically, *MsMYB1*, *4*, *5* and *12* belong to S14, *MsMYB17* to S2, *MsMYB11* and *15 to* S20 and *MsMYB13* and *MsMYB16* to S22 (Fig 5C). The function of several genes in some of these subfamilies is known: for example, the loss-of-function of *AtMYB73* (subgroup 22) is associated with the hyper-induction of the genes *SOS1* and *SOS3* in response to exposure to severe levels of salinity[44], while product of *AtMYB68* (subgroup 14) is a regulator of root growth[45]. Meanwhile, the product of *AtMYB23*, a gene belonging to the same subgroup, provides a positive feedback loop for cell fate specification in the root epidermis[27]. Given that the *A thaliana* MYBs have such a wide range of function, it is likely that the same applies for the *MsMYBs*.

## Conclusion

Alfalfa is one of the most widely cultivated of perennial forage species, so there is some research priority attached to improving the current level of understanding of the physiological and molecular mechanisms governing its stress response. Here, a comparison was made between two alfalfa cultivars contrasting with respect to their salinity tolerance, with a focus on both the physiological and the transcriptomic response to the stress. A set of 17 differentially transcribed *MYB* genes was identified, and their profile of transcription in each of the two cultivars was obtained. The open reading frames of all 17 genes were isolated, which allowed their responsiveness to salinity stress to be ascertained. When one of these genes, namely *MsMYB4*, was constitutively expressed in *A*. *thaliana*, it was shown to increase the plants’ salinity tolerance ability in an ABA dependent manner. As yet it is unclear whether or not the products of any of these *MYB* genes are involved in the regulation of abiotic stress other than salinity, nor is it clear what the nature of the mechanism underlying the response of MsMYB4 to salinity stress may be. Addressing these questions is the aim of continuing research.

## Supporting information

S1 Fig The R2R3 core domain sequences present in each of the 17 *MsMYB* sequences. (JPG)

S2 Fig Gene Ontology analysis of the DEGs identified from DL or SD seedlings grown either in the absence or presence of salinity stress. (JPG)

S1 Table Summary statistics of the six RNA-seq libraries constructed from the seedling roots of DL and SD. (DOC)

S2 Table The set of genes transcriptionally re-programmed in salinity-stressed DL seedlings. (XLS)

S3 Table The set of genes transcriptionally re-programmed in salinity-stressed SD seedlings. (XLS)

S4 Table GO enrichment of genes transcriptionally re-programmed in salinity- stressed DL and SD seedlings. (XLS)

S5 Table The set of *MsMYBs* that show differential expression in the DL and SD seedling root transcriptomes. (XLS)

S6 Table The full length coding sequence of each of the 17 *MsMYBs*. (DOC)

S7 Table The sequences of primers used. (XLS)
