# Supplementary figures and images for: Transcriptional profiling reveals that a MYB transcription factor MsMYB4 contributes to the salinity stress response of alfalfa
Source: PLoS One. 2018 Sep 25;13(9):e0204033. doi: 10.1371/journal.pone.0204033 (PMC6155508; doi:10.1371/journal.pone.0204033)

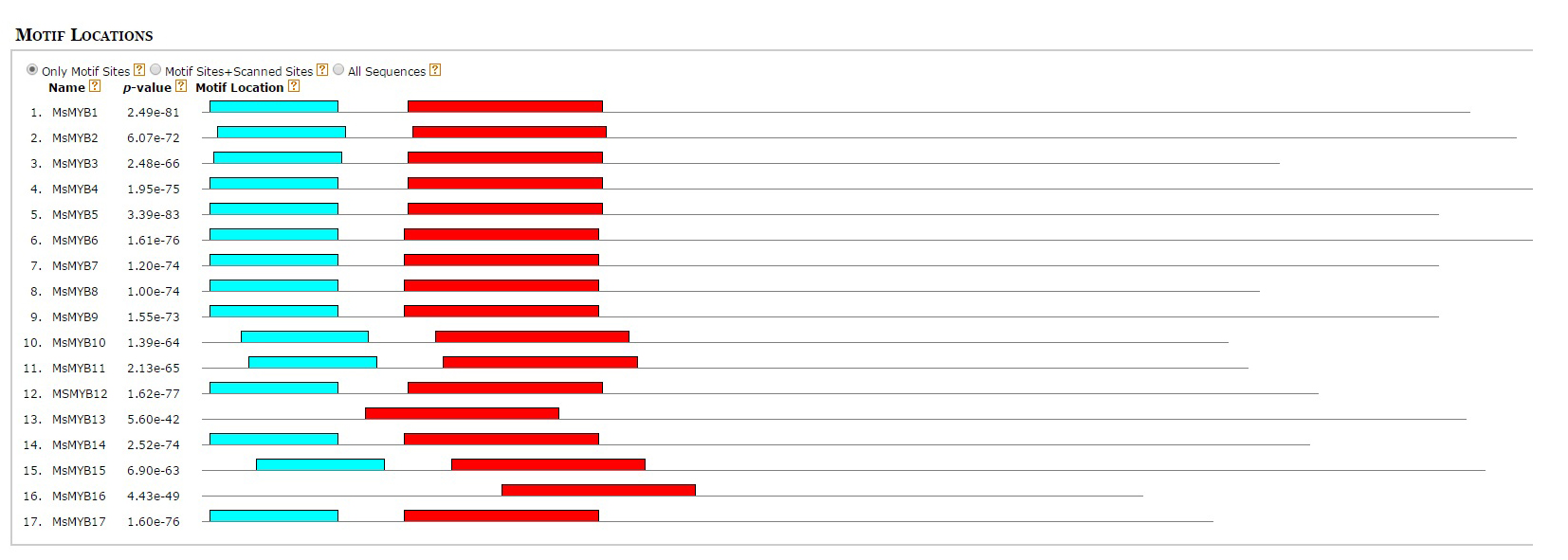

Supplement: S1 Fig — (JPG) [file pone.0204033.s001.jpg]

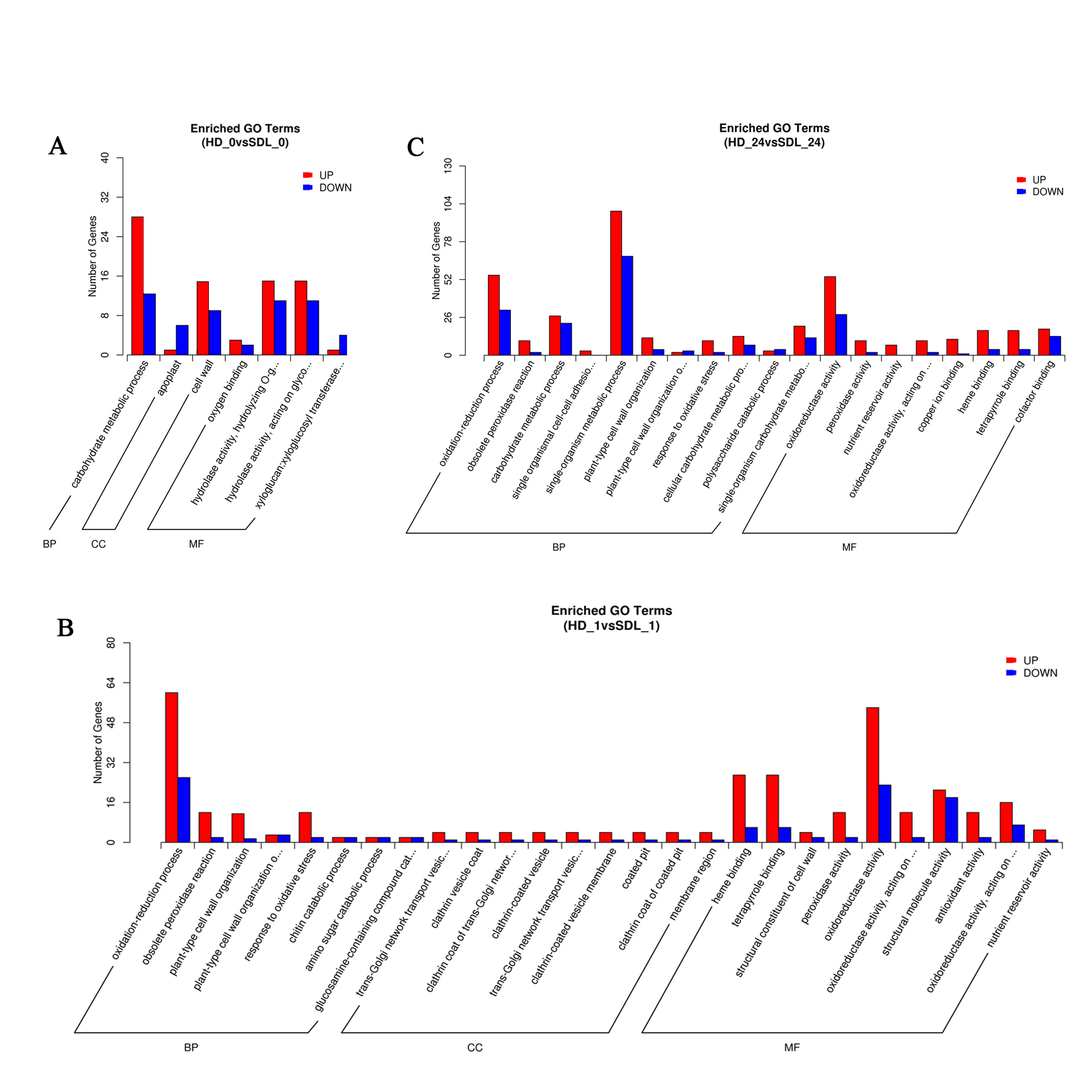

Supplement: S2 Fig — (JPG) [file pone.0204033.s002.jpg]
