# Supplementary material for: Transcriptional profiling reveals that a MYB transcription factor MsMYB4 contributes to the salinity stress response of alfalfa
Source: PLoS One. 2018 Sep 25;13(9):e0204033. doi: 10.1371/journal.pone.0204033 (PMC6155508; doi:10.1371/journal.pone.0204033)
Supplement: S1 Table — (DOC) [file pone.0204033.s003.doc]

| **Mapping to NCBI unigene databases** | **DL CK** | **DL 1h** | **DL 24h** | **SD CK** | **SD 1h** | **SD 24h** |
| --- | --- | --- | --- | --- | --- | --- |
| Raw reads | 42218844 | 54893230 | 60643490 | 44128216 | 44332690 | 44187872 |
| Total number of clean reads | 40872344 | 53288058 | 58609528 | 42273256 | 42804660 | 42698554 |
| Clean reads mapped to ref | 29087938 | 37933980 | 40984488 | 29751168 | 30074874 | 29275674 |
| % of clean reads mapped to ref | 71.17% | 71.19% | 69.93% | 70.38% | 70.26% | 68.56% |

**Table 1** Summary statistics of the six RNA-seq libraries constructed from the seedling roots of DL and SD
