# Supplementary material for: Transcriptional profiling reveals that a MYB transcription factor MsMYB4 contributes to the salinity stress response of alfalfa
Source: PLoS One. 2018 Sep 25;13(9):e0204033. doi: 10.1371/journal.pone.0204033 (PMC6155508; doi:10.1371/journal.pone.0204033)
Supplement: S6 Table — (DOC) [file pone.0204033.s008.doc]

1. *MsMYB1* CDS

ATGGGAAGAGCTCCTTGCTGTGACAAAGCAAATGTGAAGAAAGGACCATGGTCTCCAGAAGAAGATACAAAGCTCAAAGAGTATATAGAAAAACATGGAACTGGTGGAAATTGGATTTCTCTTCCTCAAAAAGCTGGTCTAAGGAGATGTGGAAAAAGTTGTAGATTGAGATGGCTAAACTATCTTAGGCCAAACATTAAGCATGGGGATTTTTCTGATGAGGAAGACAGAATAATTTGCAGTCTCTATGTTAATATTGGAAGCAGGTGGTCAATTATAGCAGCTCAATTGCCAGGTAGGACAGACAATGATATCAAGAACTATTGGAACACAAAGTTGAAGAAAAAACTCATGGCTATGCAGTTTCCAACATTACATCATCAAAGAAAACCTTCATTTCCACCTTCTCCTTCTTCTCATCAATTCTTAGACTACTTTTCTCACACACCAACTTCCTTCATAGATCTTCAAACCATTTCACTTCCTTCAAATAACTATCCAAACACAAGTTTCAACCCTATTTACCAAAATCAAGAGTCCATGGTTAGTGTTGTTAATCCTCCCATGCAATATAATTACCCTATTAAAGATAACATGTTTATGTTTGGAAGTGAAGGAAGTAGTTCATCTGATGGAAGTTGCACTCTTAGTCATGGCAAAGAAATCAAGCAAGAAGAAATTGTTTATCATCATCACATGAATAGTGGTGGATTTGAATTTGATGGCTACAACAATAAGAACAACTTTATGATAAATGGTAACAATGGAAGTATTGTTGGTGAAAGTGTTAACCAATATGAAGAAAAATCAAGTGGAGTTGGATATAGTTATAATAATGGTCAAAGTCAAATATTAAATCCATTACTAGATTATGGTCTTGATGATATTAAACAATTGATTAGTAGCAGCAGCAACAAAGGTTTTCATGTTGATGATATTCATAAGAATGAAGAGATTGGTATGTACTACTTTGATTATTAA

1. *MsMYB2* CDS

ATGGGAAAAGGAAGAGCACCATGTTGTGATAAGAGTCAAGTTAAGAGAGGACCTTGGAGCCCTGCTGAAGATCTCAAGCTTATAGCTTTTGTTCAGAAATTTGGTCATGAAAATTGGAGAGCTCTCCCTAAACAAGCAGGTCTTCAACGATGTGGGAAAAGTTGTCGTTTGAGATGGATAAATTATCTTAGGCCTGATTTGAAGAGAGGCAATTTTACCGCAGAGGAAGAGGAAACCATAATAAAGCTACATAAAACCTTGGGAAACAAATGGTCAAAGATTGCATCTTATTTTCCTGGTAGGACTGATAATGAGATCAAGAATGTGTGGAACACACATTTAAAGAAAAAACTTGTTGTCAAAAAATCAGAGTCAAGTGGAGATGAGTCCAAATTAGAATCATCAATAACCTCATCCTCTTCATCAGAATCATTTTTATCAAATGAGGTACCAATAATGAAAGATTCTGAAAAACAAATATCCAACAATGAACTTGTTATAATCAATGAGGATCCAAAAGGGTCATCATCAAATTCATTATCATCTTCAATTGAGTCTAATATCTTGAACTCAAGCCAAATTGTTAACAACGATAATTTAGAACAAGAATTGGCTTCTTTAGGGTTTTATGATGATGTTGGCAATATATTAGAAGATGTGGATGATCCAAACAATTTGATTGAAATACCATGGGAATCAGATTATGATCTTTGGAATTTTATTGACAACATTGGAACTTATCAATCAAATGTTGGTGAAGAGACTGTTCAAGATGTTGTTGAAGCAAAAAATTGGTCATCACATGATGAATTTGAGAATGAGTTTGGAGTAGTAGGTGAAACAAAGGAGTCAAACAACAAAGATGAGGTCCTACCAAAGAATTATGAAGTTGAACAAGAAATTGACCCTCATGAGGCATTTGATTTCAATGATATTATAATGCCAGATTCTGAATTAGATTTTGGTAATATTCAATTGTGGCCCTCTTTGCCTCAAAATGGTATTCCAAAAGTTTAA

1. *MsMYB3* CDS

ATGGCTAGAACTCCTTCATGTGACAAAATTAGTGGAATGAGGAAAGGGACATGGACTGCTGAAGAAGATAGGAAATTAATTGCTTATGTCACTAGATATGGTTGCTGGAATTGGCGACAACTCCCTAAGTTTGCAGGTCTTTCAAGGTGTGGGAAGAGTTGTAGACTTAGGTGGCTGAATTATCTTAGGCCTAATATCAAAAGAGGAAACTTTACTCAACAAGAAGAGGAGTTGATTATCCGAATGCATAAAAAGCTTGGGAATAGATGGTCAACCATTGCGGCTGAGTTACCGGGAAGAACAGATAATGAAGTGAAGAACCATTGGCACACTTCTCTCAAGAAGAGAGCTATAGATAACATAGTCACGAATGAAGAAACGAAATCAAAAGACATAATTGAATCGACACAAGGGAGAGATATATCCAATTACCAAATTACTCCTCAATCCAGTTCCCAAATCTCAGACACCAATGGCCCTTTATCCCCATTTTCATCATCTAGTGAAATCACTTCCACATCTTTAGATCACAATTCCACTTCTTTGGATGATTTTGGTTTTCTAGATTCATTTATAGAAGATGTGGATGAAAGTTTTTGGCTTCATAATCTTTCCAACACACCAAGTGGAACAGTGCAAAACCATACTACCGAAAGTTTTGTCATGGATAATGATTTTGGAAGCTTTTTAGATGCTTACAAGGAGTCAGCAGTTGATAGTTTTTGGACACAACCATATGAGGATGACATGTCCCACGTTCCAACACAATTGCTGACATCTTTGCCGTTGGAATCTGAATATTTTTCTATAGTATATGATGATATTTGGAGTTGA

1. *MsMYB4* CDS

ATGGGTAGAGCTCCATGTTGTGACAAGGCTAACGTGAAGAAAGGACCTTGGTCTCCTGAAGAAGATGCTACACTCAAATCCTACATTGAAACAAATGGAACTGGAGGAAATTGGATTGCTCTTCCTCAAAAAATTGGGCTCAAGAGATGTGGAAAGAGTTGCAGACTTAGGTGGTTAAATTACTTGAGACCTAATATCAAACATGGTGGATTTACTGAAGAAGAAGACAACATCATTTGCAGCCTTTACATAAGCATTGGAAGCAGGTGGTCCATTATTGCTGCTCAGTTACCTGGAAGAACAGACAATGACATCAAAAACTATTGGAACACAAGATTGAAGAAGAAATTACTCGGAAAGCGAAAACAATCGAATAACAACAACAACTTGATGAATCAAAAGGACACAAATGGAATAGATGATAATTCTTATTCAAATTCATTAAGTAGCTCAGCTCTTGAGAGACTTCAACTTCATATGCAACTTCAAAGTCTTCAAAACCCTTTGTCTTTTTACAATAATAACCCTGCTGCACTAGTTTGGCCAAAGTTGCATCCTTCTCAAGAAAAAATGATCCAAATTAGCCTTCAAAACTCTAATAACAACCCTATGATGCAAAATGCTTTCTCTTCACCACAGGTTGATCTTTTGGAGACTATTATTCCTTTGGAGAATAACAATACTTCAGTTACCTTCAATGCTTCTGGAAATAGCAGTAATAATAATAATTCAATCATGCATTCAAGTGTTGCACCAAGAGGAGAAGCTGTTGAGAAGAGTACTAACAATGAAGGAATTCAGGAACTGGAAAGTGAACTAGATGAAATTCTCAATAACAGAAATATAATTACACTGGAAGATGAATATCGTGTGGCTGAATTTGATTGTTTCAGAGATATGAATAACAATGGTTCAAAGGATCAAAACTTGATATGGTGGTCAAATGATTCAGGTGATACTAAATCAGGATCCTCAAACTCATGGGATTCATCAACTAATCTTATGCAAGAAGGGATGTTCCAAGATTATGAACTAGGTTATGGTCTGTAG

1. *MsMYB5* CDS

ATGGGAAGAGCTCCTTGCTGTGACAAAGCAAATGTGAAGAAAGGACCATGGTCTCCAGAAGAAGATACAAAGCTCAAAGAGTATATAGAAAAACATGGAACTGGTGGAAATTGGATTTCTCTTCCTCAAAAAGCTGGTCTAAGGAGATGTGGAAAAAGTTGTAGATTGAGATGGCTTAACTATTTAAGGCCAAACATTAAGCATGGAGAATTTTCAGATGAAGAAGACAGAATCATTTGCACTCTTTATGCTAATATTGGAAGCAGGTGGTCAATTATAGCTGCTCAATTACCTGGAAGAACTGATAATGATATAAAAAACTACTGGAACACAAAACTAAAGAAGAAACTCATGAATCTTCTTCCTCAATCTCATAACACAGTCTTACCATATCTAACTTCTTCTTCATTTCCTTCTCATTGCACCTCCTATTACAACCAAACACCAACAAACACATCTTTCACAACATCCCTTGAACAACAATTTTCTGTTCCTTCATCAAGTAGCTCAATCTCTCTCCCTTTTTACCATAACCAAGAGTCCTTACTCAATGGATTTAGTAGTAATACTAATACTTCCATGCAATATCAACTTCAAAACCCTATGATGAAAGAAAGTCTTGTCATGTTTGGAAGTGAGGGAAGTTGTTGCAGTTCATCTGATGGAAGTCTTGGGAAACAAGAAGAAATAATGGGGTTTCAGAATTTCATGCAAATCAACAAGTTCAATCTTAGTCATGGAAGTGATGTTGTTGATGTTAACCAATGGGAAAGAGAAAAGGTGAATTTATGCTTTAGTCAAAATCATGAAAAGCAAATTACTAGTACTACACCTTTGGATTATGATCTTGAGTATATTAAGCAATTAATTACTAGTAGTAATAGTGGTAGTTGTAATAATGGTTACTTGAGTATTGATGAAAACAAGATGGAGGAGAAGACTATGTACTATTACTGA

1. *MsMYB6* CDS

ATGGGAAGGTCCCCATGTTGTGATGAGAGTGGCCTAAAGAAAGGACCTTGGACTCCTGAAGAAGATCAAAAGCTAGTTGAACACATTCAGCAACATGGCCATGGAAGTTGGAGAGCACTCCCTAAGCTAGCTGGTCTTAATAGATGTGGAAAGAGTTGTAGGCTAAGGTGGACCAATTATTTAAGGCCTGATATTAAGAGAGGAAAATTTTCTCAAGAAGAGGAGCAAACAATTCTTCATCTTCATTCAATCCTTGGAAACAAATGGTCAGCAATTGCCACACATCTACCTGGAAGAACAGATAATGAGATAAAGAATTTCTGGAACACTCATTTGAAGAAAAAGTTGATTCAGATGGGTTTTGATCCAATGACTCATCAACCAAGAACTGATCTTGTTTCCACCATACCATATCTACTAGCTTTAGCAAACATGACAGAACTTATGGATCATCATAATCAGTCATCATGGGATGATCAACAACATGCTGCAATGAGTAGTTTACAAGCAGAAGCAGTTCATCTTGCAAAGTTTCAATGCCTTCAATATTTACTCCAATCTTCAAATTCTTCTATAAACATTAATAATAATTCCTATGACCAAAATGCCATGATCACAAACATGGAACAACAACAACCCTTAAGTTTATTAAACACAATCTCTAATGTGAAAGAGAACATAATAATGGACTGTTCACAACTGGATAGTACTCATGCAGTCTCATTTTCTCAACCACTCCACCACCAAAGTGTGCTACCCCACTTTTTAGACCCACAACAAGTCTCTTTCAGTTCACAATCATGTTTGAATAATGAACAAAGTCAAGGTGGTACTATTACCAACTTTGCAACAGTTGATGAAACATCATCATGGATTAATGTTCCATCTTCTGCTCCAATATCTGTTCCTCTAAATGCTATGGGGATTTCAGCAGGAGATGCAAGCAGCTGCACTTCTAGCTATGGAGGAGGAGGAGGAGGACCCTCTCCAGTATCTTATTGGTCTGAACTTTTTTTTGAGGACCCCATTATGCATGATTTATCTTAA

1. *MsMYB7* CDS

ATGGGTAGATCTCCTTGCTGTGAAAAACAACACACAAACAAAGGTGCTTGGTCCAAGGAAGAAGATGAACGCCTTATTAACTACATCAAACAACAAGGTGAAGGTTGCTGGCGTTCTCTTCCTAAAGCCGCAGGCTTGGCAAGATGTGGAAAAAGCTGTAGATTGAGATGGATCAATTATCTCAGACCTGATCTTAAACGTGGAAACTTCACTCACGAGGAAGATGAACTTATCATTAGCCTCCACGCTATGGTTGGAAACAAATGGTCTCAGATTGCTCAAAAATTGCCCGGAAGAACAGACAATGAGATCAAGAACTATTGGAACACTCACATAAAACGCAAACTCTATAGCCGTGGAATTGATCCTACAACACATCAGCCACTCAAAACCCTCTCCGGCGCCGCTGCCGCTTCAGCCGGTACTTCCTCAGCAACAACCACCTCTGCAGAAGCTGCTTCTCCTTCAATGGTTGTTAATAATAATTCATCCGTATTACCTGTAGTACCCTCAACCAACGAGAGCAGTACTAGTAGCAGCATGAGCCACCACCACAACAAGATTAAGAGTGAGTTTCAGTTATTCAGTAATTTCAGTGGCTCTCGGCTTAAAAAGTTTGTTGCAACTGATTCTGTCCATGGTGGTGTTGAAGAATCAGTTTCAAACAGTAGCAGTGGTGTTACCGTTGAGGATATAGCTTATCCTCCTCCAATCAACTTGGAACTTTCATTGGCTATATCCTCTCCCTCTCAACAGCCACAGCAAAAAGCTTTGTGTCTTTGTCGTCAAATATCTTTAGGGTTACATGGTAGTAGCCACCAACCTTGTTGCTGCAATAATACCATGCCTATCGTTGGTGTTTCAACTTCTTATGCTGTTACTGCTCCACCACCTGCTACCACCGGCGTCGGGAATGGTTTTTTCAAATTTTCCGGGGCCGGGATTGTCAAGTTTTGA

1. *MsMYB8* CDS

ATGGGGAGACAACCTTGTTGTGACAAACTTGGTGTGAAGAAAGGTCCATGGACAGCTGAGGAAGACAAGAAACTCATCACTTTTATTCTTACCAATGGACAATGTTGTTGGCGTGCTGTGCCTAAACTTGCCGGCCTTCGGCGCTGCGGTAAGAGCTGTCGTCTTCGTTGGACTAATTATCTTCGTCCTGATTTAAAGAGAGGTCTCCTCACTGAAGCAGAGGAGCAGCTTGTTATTGATCTCCATGCCCGCCTCGGCAACAGGTGGTCGAAGATTGCTTCACGGTTACCCGGGAGAACAGACAATGAGATTAAAAATCATTGGAACACACACATCAAGAAAAAGCTCATTAAGATGGGAATTGATCCCATTACTCATGAACCTTTGAATAAACAAGCATCATCCAATGATAGTAGTACTTCATCCCCTGCAGAAAATTCGTCACAGCCTGTTAACAACCATGAAGTGAAGGAAACTGATGGGGTTGTCAACTCAGAGGAGAATTCAAGCTCATCCCCAGCTGAAAATTCTTCTGGAGAAGAATCACTTTTGCTAGATAGCATTTGCAGTGACGATTCTCTAATGAATAGTATATGGTTGGATGAAACACCATTGGTGGAAGCCCTTTGGGAAATGGACACTACACCTATAGCAGAAAACACAAAAAATGACATGAGTTTTATGCCTTCTTGGGAGGATAATTGTGCTTGGCTATTTGATTGTCAGGACTTTGGTATTCATGACTTTGGATTTAATTGTTTCAATGAGATAGAGTCAAGTACACTGCAAACTATAGGGATGAAGGAAAATAAACATTAG

1. *MsMYB9* CDS

ATGGGAAGACAACCTTGTTGTGACAAAGTTGGATTGAAGAAAGGACCATGGACTGCTGAGGAAGATAAAAAGCTCATCAATTTCATCCTTACTAATGGTCAATGTTGCTGGAGAGCTGTCCCTAAGCTAGCAGGGTTGTTAAGGTGTGGAAAAAGTTGCAGATTGAGATGGACAAATTACCTAAGGCCAGACCTAAAAAGAGGACTTTTATCAGAATATGAAGAAAAAATGGTCATTGATCTACATGCTCAACTTGGCAATAGATGGTCTAAAATTGCATCTCATCTACCGGGACGAACCGACAATGAGATTAAGAATCATTGGAATACTCACATAAAGAAAAAGCTCAAGAAAATGGGAATTGATCCAGTTACACACAAGTTACTCTCTAATGCAACTATTGAGCAAACTCAAACACAATCTGAACAAGAACCTCAACAATCTTCTTCACCTATTGAAATGGAACACAATGTTGAATTTGAGAACCACAAAAACAAGGAACCACAAAAACCAGAAACATCATTTGAGTCATCAACAATAACTGAAGCTAAAGAACAAGACCAAATTATGACACCACTTTTTGACTCAACAATGGAACTAATGAATGAGTTATTCACTGAAGAAGTTCCAATAATAATACCAAATGAAATTCTAGTTCCATGTGTTCCTTCATCTTCATCAACCACAACATCAACATCAACATCATCAACTTCAAATTCAAATTCATCTAACTTCCTTGAAGACTTGCTTCTCCCTGATTTTGATTGGTCACATGATAATAATATTGAAAATAATAACTATAATGGAAATAGTGACAATAATAATAATATTAATATGACATTATGGGATGATGACTTTATTAGAAGTTGGGATTTTGTGATCAATGATGATGATGATGGTGACAAGGATTCAAGAGTGATCATGGATTCAGAATCATGGGCCTATGGATTATTTTGA

1. *MsMYB10* CDS

ATGTATTCAGGAATGATGGAAGGAAACACTGGATGGAGTGTAATGGAAGAAGATAGATGGAGGAAAGGACCTTGGACTTCTGAGGAAGACAAATTACTCATTGAGTATGTCAAGCTGCATGGTGAAGGCAGATGGAACTCTGTCTCTAGGCTTGCAGGACTGAGAAGAAATGGGAAAAGTTGTAGACTGAGATGGGTGAACTACCTAAGACCAGACCTTAAGAAGGGTCAGATAACACAACAAGAAGAAAGCATAATCCTAGAGCTACATGCTAGGTGGGGAAACAGGTGGTCAACAATTGCAAGAAGCTTGCCGGGAAGAACTGACAATGAGATAAAGAACTATTGGAGGACTCATTTCAAGAAAAAGACCAAAAACCCCTCTGATAGTGCTGAAAAGGCGAAAAATCGTTCTTTCAAGAGGCAGCAACAACAACAATTGAAGAAACAACAGCAACAAGTTCAGATGCAGCAACAACAACTGCAATACAACATGGATATGAAAGGGATCATAGACTTGTTGCTTGAGGAAAATGACTACTGTACTAGTGTGCCTTCTACTTCTCAAGAGACACAAGAAATGGTTTCCATGTATGCTGATACACAAGAACAACAGGGTTGCTTTTATTCTATGCTCAATGATAATAGTGGTAATGTCTATGCACAAGAGTCTTCAAATGAAGAGAATTTGTGGGATGAACTTTGGAACTTGGATGATGCTCTTGGAAATTCAATGCAGCTAATGCTTCAAGCAAAGCCAGCCAATGTGCACAATGTAGTTGCTCCCTTTTGTTAA

1. *MsMYB11* CDS

ATGAGAGGTATGGATATTATTAAGGTTCAGAAAGGTGGGTCTGCAAAGGAAAATGAGACAGGGTTAAGAAAAGGTCCTTGGACATTAGAAGAGGACACCATTCTGGTTGATTACATTACAATACACGGTGAAGGTCACTGGAATACCCTTGCATCTTCTGCAGGTTTGAGGAGAAGTGGTAAAAGTTGCAGATTAAGGTGGCTAAACTACTTGCGTCCCGATGTACGCCGCGGGAATATCACAGTTCAAGAACAGATATTGATTCTTGACCTCCACTCTCGCTGGGGCAATAGGTGGTCGAAAATTGCACAACATCTTCCGGGAAGAACAGACAATGAAATAAAAAACTATTGGAGAACAAGAGTGATCAAGCAAGCAAAACAGCTCAAGTGTGATGTCAACAGCAAACAATTCAGAGACGTTTTACGTCACGTTTGGATGCCCCGATTGCTCGAACAAATTCAGCCCGCACAACAATTCCCCGACACGAATAATCCAAACGGATCAAATATCCTTCTTCAACAAAACACATTGCAAAGTTCAGTTTCAGGAATCAGTGGTGTTTCCTCGGACTCTTCATCAGTAGAATTCCAAGTTGCTTCAAATTCGGACAAAAATAATTCTTTGGAGCTTTTAGGCCATGAAGGTTCAAAACCATGGTCAAGTTTCAACAACCAGGTTTCAGAACAGGGGAAGAGTACTGGTGCTTGTGATGGTGACTCGTTGGAGAGTATGTGGAACGATGAGAACATGTGGTTTTTGCAACAACTTTACGAAGATGTTGAAATAAAATACAATTTACTTGCGTGA

1. *MsMYB12* CDS

ATGGGAAGATCTCCTTGCTGTGACAAAGCTAATGTAAAGAAAGGTCCATGGTCTCCTGAAGAAGATGCAAAACTAAAGGAGTACATAGAGAAACATGGAACTGGAGGAAACTGGATTACTCTGCCAAAAAAAGTTGGTTTGACAAGGTGTGGCAAGAGTTGTAGACTTCGATGGCTTAACTATCTCAGACCAAACATTAAGCATGGAGAGTTCTCTGACTCTGAAGATAAAATAATATGCACCCTCTTTGCTAGCATTGGAAGCAGGTGGTCAATAATAGCATCTAAGTTAAAAGGCAGAACTGACAATGATGTAAAGAACTACTGGAACACCAAGCTTAAGAAAAAGATTATGGCCATGAATCATTCTCATTCCGCGGAGATGAAACCTCAACAAGTTACCCTTTTATCCATCCTTCAAAACTCAACAAAATCATCTCCATCATTATCATTCACAGACAACTCATACGATGATCACTCTAATGGATCTTTCAGTACAGGTTCAATCTCCTACTCATCTACATCATCAAGTCTTTTGAGTGGAAATTCTTCTACTTCTGCAGCACAAGAAAGTTTCATTAGTCCCACTAAGAGTAATAGCAAAAACCAAATCAGCCATGTCATAGATCAAGAGCTTGTTGAAGGAAATGGTGATGCAACTTTTGTAGAGCAGAATAATATTGATTCTGTTGATTACATCTACAGTGAGGTGGAAGATAGTGAGAAGTTAATGTTCTCTAATGCTGGTAGTGTTAATGGATTATGGGGAGAAAATCCATTGGATTATGATATAGATGAAATTAAACAGCTAATAAACACTACTGCATCTACTAGTAGTTGCAACAACTTTTTGTTTGTTGACTAA

1. *MsMYB13* CDS

ATGGATCGGATCAAAGGTCCATGGAGTCCAGAAGAAGATGAAGCACTTCAAAAACTCGTGGAGAAGCACGGGCCGAGAAACTGGTCGATTATAAGCAAATCGATACCGGGCCGGTCAGGGAAATCATGCAGATTACGTTGGTGTAATCAGCTTTCACCACAAGTTGAGCATCGGGCTTTTACGCCTGAAGAGGATGATACTATAATCAGAGCCCATGCTCGATTTGGAAACAAATGGGCTACAATAGCCAGGCTTTTATCGGGCCGAACTGATAACGCTATTAAGAATCATTGGAACTCTACTTTGAAACGTAAGTGTTCTTCTATTATGATTGATGATTCTGAATCTCCACCGTTGAAAAGATCTGTTAGTGCTGGTGCTGCTATTCCTGTTTCTACTGGTCTTTATATTAACGCCAATTCTCCTGGTTCTCCTTCTGGATCCGATGTGAGCGAGTCCAGCGTACCTATTGTGAACACTCATGTTAACTCTCATGTAAACTCTCATGTTTACCGACCTGTGCCAACGAGAACCGTCGCGCCTCTACCACTCGTAGAAACGACGTCGTCTTCCAAATCTTCGAAATCGAACGATCCTCCAACTTCGTTATCACTCTCACTTCCCGGTGTTGGTGTTGGTGTTGATTCTTCTTCTGAAGATTCAAACCGTGTAACTGAGCCTGTCAACACTGTACCACCACCTCCTCCGCCACCACCGCCAAGTGCGATCCCTTTGATTCCGGTAATGACTACGGCTGTTCCTGTTCCGATGGCGGTTCCGATGCAACAATCGGGATCAGTGATGCCATTTAGTTTTAGTGCGGAGTTGTTGAGTGTGATGCAGGAGATGATTAGAACAGAGGTGAGGAGTTACATGGCGGGATTGGAGCAACAGAATGGGATGTGTTTTCAAAAAGCTGAGGATGGAATTAGGAATGCTTTGGTGAAGCGAATTGGAATTAGCAGAATCGATTCGTAA

1. *MsMYB14* CDS

ATGGGAAGATCACCTTGTTGTGAAAAAGCTCATACAAACAAAGGAGCATGGACAAAAGAAGAAGATGATAGACTTATATCATATATTAGGGCACATGGTGAAGGTTGTTGGAGATCTCTCCCTAAAGCAGCTGGCTTACTCCGATGTGGTAAAAGTTGCCGTCTCCGGTGGATTAACTATCTCAGGCCAGACCTTAAACGTGGTAACTTCACAGAAGAAGAAGATGAACTCATCATCAAACTCCATAGTCTTCTTGGTAACAAATGGTCTTTGATAGCTGGAAGATTACCTGGAAGAACAGATAATGAGATAAAGAATTATTGGAACACTCATATAAGAAGAAAGCTTTTGAATAGAGGAATTGACCCTGCTACTCATAGGCCTTTAAACGAAGTTTCTCATTCTCAATCTCAATCACAATCTCAAACTCTTCATCTTCAAAATCAAGAAGCTGTTACTATAGCTGTAGCAGCATCTACATCAGCTCCCACTGCTACAAAAACCCTACCAACAACTATATCTTTTGCATCATCCATCAAACAAGAACAATATCATCATCATCATCAAGAAATGAACACAAACATGGTTAAAGGGTTGGTTTTAGAACGTTGTCCTGATTTGAATCTTGAGCTAACAATTAGTCCACCACTTGTTCAAGAACACGATGAACAATTCAGAAACAGAGAGAGGAACAATCTCTGTTTTGTTTGTAGTTTGGGTTTGCAGAATAGTAAGGATTGTACCTGTGATGAAATTGTTGGAAATTCTAGCAGTGGAAATGGTTCTACTGCACCTGCTTATGATTTCTTGGGTTTGAAAGGTGGTGTTTGGGATTACAAAGGCTTAGAAATGAAATGA

1. *MsMYB15* CDS

ATGGAAGTGGAAGGAAGTGTAATAAGCAACTCGAACATCACCATGCTCCAAAGTGATGATGAGTTGGATCTTCGAAGAGGACCATGGACCGTCGATGAAGATCTTGCTCTCATCAATTACATTGCAAATCATGGTGAAGGTCGATGGAACACTCTTGCTCGTTCAGCTGGACTCAAACGAACTGGCAAGAGCTGTAGATTGAGATGGTTGAATTATCTTCGTCCAGATGTTCGTCGTGGTAACATCACACTTGAAGAACAACTTCTTATTCTTGAACTTCATAGTCGTTGGGGAAACCGATGGTCCAAAATTGCACAATACTTACCGGGAAGAACAGACAACGAGATCAAGAATTATTGGAGAACCCGTGTCCAAAAACATGCCAAACAACTCAAATGTGACGTAAACAGTAAGCAATTCAAAGATGCCATGCGCTACCTTTGGATGCCAAGGTTAGTGGAGCGCATCCAAGCTGCCGCATCCACCTCCGCCACCCGAGCCACCGCCACTAATAATGGTTCACCAAGTGGTACAACAGCGGTCACCACCAACAATACGGCATATAACATAAACAACAATTTTGATGTTCATGGTAGCGCAAACATGTTAACTCCAACAATTATGAACACCAACAGTTTATGTGGTTCACATGTTACACAAAGTTATACTCCGGAAAATAGTAGCAATGGAGCCGCGGCTGCATCATCGGATTCATTTGGGACACAAGTTTCGCCTGTCTCGGAATTAACTCAAGATTATTACAATGTCCCCGTTGGTAGTAACAACAATAATAACCCTAATTCTGATTATTACCAACAAGCTCAACAATTTGGGTTTTTTGATTGCATCACTAGCCCATCTGGATTGTTCCCTCAAATGGATTTCAATTCCATGGAACCAAACACTCCTTGGATTCAAGGGGACACATCTGACAATTTCTGGAATGTTGAAAACATGTTGCTCTTTCAACATCTCAATGACAACATCATGTGA

1. *MsMYB16* CDS

ATGAATATGAATATGAATTTTGAAGCTATGAATAGAAGCTCTTCAACAGCAACATCATCATCAGACTCATGTTCCTCAGAATCAAACCCAACAAACCAAAACAAACTAGACAGAATCAAAGGTCCATGGAGTGCAGAAGAAGACAGAATTTTAACACGACTGGTTGAACAACATGGAGCAAGAAACTGGTCACTCATCAGCCGCTACATAAAAGGAAGGTCAGGAAAATCTTGCAGACTCCGGTGGTGTAATCAGCTGAGTCCCACGGTGGAGCACCGTCCATTTTCTTCACAAGAAGACGAAACAATCATAGCGGCACATGCTCAATATGGTAACCGATGGGCTACCATTGCAAGGCTTTTACCTGGTAGAACAGATAATGCTGTCAAGAATCATTGGAACTCTACGCTTAAGCGTAGAGCGGGTTGTGGCGCTGGTCGTGGTGTTTGTGGTGGTTCTGTTACGGTTGCTGGAGGTGTTAATGGTGGTAACGAGGGTGGAAATTGTTCTGGTCAGTTTGCTTTTCAGGTGGAAGATGATCCGTTGACTGCGTTGACTCTTGCTCCTCCGGGGAATGGTGTGGAAGATAGGGAGGAAATGGTTGGTGATCATCGGTTTCCTTCACCGTCGGTGTCGCCGGAAAATGTTCCGACTGAGTTTTGGGATGTGATGAGAGGTGTTATTGCAAGAGAAGTTAGGGAATATGTTTCTTCTAATTTTTCTCATTAA

1. *MsMYB17* CDS

ATGGTAAGAGCTCCTTGTTGTGAAAAGATGGGATTGAAGAGGGGTCCTTGGTCTCTTGAGGAAGATGAAATCCTTACATCTTACATCCAAAAACATGGCCATGGCAATTGGCGTGCCCTCCCAAAGCTTGCAGGCTTGTTAAGGTGTGGGAAAAGCTGCAGACTTAGGTGGATTAACTATTTGAGACCAGATATCAAGAGAGGAAATTTCACAAATGAAGAAGAGGAAAATATCATTAAGCTACATGAAATGCTTGGAAACAGGTGGTCGGCAATTGCAGCAAAACTACCGGGAAGAACGGACAATGAAATAAAAAATGTGTGGCACACGCATTTGAAGAAGCAGCTAAACCAAACAAACTCAGAAACAAAGAAAAGGGTAATCTCAAAACCAAAAATCAAACGTTCTGATTCAAATTCAAGCACAATAACACAATCAGAATCAGCATCTGCATGCACTACAAGTTCTAGTGATTTTTCATCTGTCACTATTGGTGAAAGAAAGGATATAAAAAGTGAAGACATTGAGTCCATGGAGGAGACAATGGCTGTAATTGATGAGAGTTTTTGGTCAGAAGCAGCAGCATTGGATGAAACTTCAACTGATATGAAATCAAGTTCTCCATTGACAATCTCAAATGAAATGTTGCCACTTCAATACCCTGATGAAATTCTTCCAGAGAGTACTCATGAAGATTATAATTCTAACTTGGATGATGGCATGGATTTTTGGTATGATATATTTATTAGGACTGGAGATCAAATAGAATTGCCTGAGTTTTGA
